# Death from Acouite

**Published:** 1846-03

**Authors:** 


*Death from Aconite.*—A melancholy event, which has recently occurred to a respected member of our profession, forcibly sets forth the danger, not only of those who incautiously undertake the management of severe disease existing in their own person, but also of the venturing, without sufficient care, on the administration of powerful medicinal agents, with the operation of which we are but imperfectly acquainted. Dr. Male, of Birmingham, recently fell a sacrifice to these practices. He had been reading a work in which was recommended a new and powerful agent (aconite) for the removal of deep-seated neuralgic pains, and having been suffering of late from an affection of that kind, which had resisted the ordinary means for its removal, he was induced to try upon his person the powers of the remedial agent recommended. Not sufficiently mindful of his age, Dr Male took the tincture of aconite in doses, the accumulation of which produced an alarming depression of the nervous system, from which he was ultimately unable to rally, and thus fell a victim to that want of a due appreciation of the circumstances of his own case, so common, we may add, amongst medical men when treating themselves, combined with the incautious use of a powerful drug, with the operation of which he was but imperfectly acquainted. This unfortunate case should prove a warning to every medical practitioner, as well in the pursuit of his professional avocations, as in inducing him, when himself suffering under serious illness, to have recourse to the advice of some brother practitioner.—*Provincial Medical Journal.—Boston Med. and Surg. Jour.*

